# Diagnostic value of aMMP‐8 and azurocidin in peri‐implant sulcular fluid as biomarkers of peri‐implant health or disease

**DOI:** 10.1002/cre2.883

**Published:** 2024-06-09

**Authors:** Vithleem Xanthopoulou, Ismo T. Räisänen, Timo Sorsa, Dimitrios Tortopidis, Dimitra Sakellari

**Affiliations:** ^1^ Department of Preventive Dentistry, Periodontology and Implant Biology, Dental School Aristotle University of Thessaloniki Thessaloniki Greece; ^2^ Department of Oral and Maxillofacial Diseases, Head and Neck Center University of Helsinki and Helsinki University Hospital Helsinki Finland; ^3^ Division of Periodontology, Department of Dental Medicine Karolinska Institutet Stockholm Sweden; ^4^ Department of Prosthodontics, Dental School Aristotle University of Thessaloniki Thessaloniki Greece; ^5^ Present address: Aristotle University of Thessaloniki Agiou Dimitriou 54124 Greece

**Keywords:** aMMP‐8, azurocidin, peri‐implant disease, peri‐implant health

## Abstract

**Objective:**

The objective of this study was to investigate the effectiveness of testing for active matrix metalloproteinase‐8 (aMMP‐8) by a quantitative point‐of‐care (PoC), chairside lateral flow immunotest and azurocidin, in the peri‐implant sulcular fluid (PISF), as biomarkers for the presence or absence of peri‐implant diseases.

**Background:**

Current research indicates that proinflammatory cytokines and extracellular matrix‐degrading enzymes may be of value to diagnose and predict peri‐implant disease initiation and progression, but more data are needed.

**Methods:**

Eighty patients with implants were recruited. PISF samples were collected and quantitatively analyzed for aMMP‐8 (chairside) and azurocidin with ELISA. Radiographic assessments and clinical indices (probing depth, probing attachment level, bleeding on probing, and plaque) were recorded after sampling. Kruskal‐Wallis test and pairwise post hoc Dunn‐Bonferroni test were used to relate aMMP‐8 levels and azurocidin levels to clinical parameters. The diagnostic ability of aMMP‐8 (ng/mL) and azurocidin was analyzed by receiver operator curve analysis. Area under the curve (AUC) was calculated and the Spearman's rho, and the coefficient of determination (R^2^) were used to calculate the correlations between aMMP‐8, azurocidin, and periodontal parameters.

**Results:**

Statistically significant differences were observed for aMMP‐8 levels but not for azurocidin between healthy implants, implants with mucositis, and those with peri‐implantitis (13.65 ± 7.18, 32.33 ± 21.20, and 73.07 ± 43.93 ng/mL, respectively), (Kruskall–Wallis test *p* < .05). The aMMP‐8 test with a threshold of 20 ng/mL has a sensitivity of 71.7% and a specificity of 77.8% to identify peri‐implantitis and healthy implants, respectively. AUC was found to be 0.814, and the accuracy of the method reaches 73.8%. Above a cutoff value of 33.7 ng/mL of aMMP‐8, the accuracy of the test to detect peri‐implantitis reaches 77.5% in relation to 62.5% of BoP from the same site.

**Conclusion:**

Taken collectively, present data indicate that the aMMP‐8 PoC lateral flow immunotest can be a beneficial, adjunctive diagnostic quantitative tool for real‐time screening for peri‐implant diseases.

## 1 INTRODUCTION

Dentists are widely using dental implants for patients' oral rehabilitation. Although, implants are popular among patients and clinicians, often, complications appear, the most significant being peri‐implantitis (Schwarz et al., 2018). According to the 2018 classification of peri‐implant conditions, peri‐implantitis is defined as a pathological condition around dental implants characterized by inflammation in the peri‐implant mucosa and progressive bone loss (Lindhe & Meyle, 2008). On the contrary, peri‐implant mucositis is characterized by inflammation in the implant surrounding mucosa without concomitant bone loss (Lindhe & Meyle, 2008). According to a recent meta‐analysis, at the implant level, the prevalence of peri‐implantitis is 10%–30%, and at the patient level, it is 20% (Derks & Tomasi, 2015).

Clinical and radiographic recordings are the main procedures for the assessment of peri‐implant conditions, but these parameters can detect the disease only in the established stage and do not detect early pathological alterations (Berglundh et al., 2018; Gul et al., 2020; Sanz & Chapple, 2012). Therefore, biomarkers may, eventually, assist to the diagnostic procedure, by detecting or defining the stage of periodontal destruction, if present (Deng et al., 2021; Sorsa et al., 2020a, 2022). Accurate diagnosis, especially at the subclinical level, is crucial for prevention of peri‐implant diseases with the anticipated increase in the treatment demand with implants, until 2030 (Sanz et al., 2019). Key biomarkers which are linked to peri‐implant inflammation can be used by the clinician to assess the onset of periodontal breakdown, as well as to predict future disease and monitor the treatment outcome (Gul et al., 2020). For example, if subclinical inflammation is accurately detected by a biomarker, although clinical signs of disease are absent, the clinician and the patient can modify the clinical protocol of supportive treatment to ensure peri‐implant healthy conditions.

Inflammatory mediators such as cytokines, chemokines, and matrix metalloproteinases are known to be involved in regulating and promoting peri‐implant and periodontal conditions and therefore are suitable candidates as biomarkers.

In fact, relevant studies have consistently shown that markers of bone homeostasis such as RANKL and OPG and host‐derived enzymes for extracellular matrix degradation such as MMPs, TIMPs, and cathepsins display differences among various peri‐implant conditions (Alassy et al., 2019; Kalsi et al., 2021). Until now, differences in methodology, subject sample, and the definition of peri‐implantitis are main obstacles for extracting useful conclusions for clinical praxis. The introduction and endorsement of the new classification of peri‐implant diseases (2018) will certainly assist to globally detect and evaluate biological complications around an implant.

MMP‐8 is released from neutrophils by their degranulation and is responsible for collagen type I degradation (Arakawa et al., 2012; Schmalz et al., 2019) in its active form. Active matrix metalloproteinase‐8 (aMMP‐8) has been investigated in the literature as a biomarker of the breakdown of both periodontal and peri‐implant tissues (Alassy et al., 2019; Al‐Majid et al., 2018; Arakawa et al., 2012; Räisänen et al., 2019; Rathnayake et al., 2017; Sanz et al., 2019; Sorsa et al., 2016, 2017). Positive correlations have been found between bleeding on probing (BoP), probing pocket depth (PPD), probing attachment level (PAL), and elevated levels of aMMP‐8 in oral fluids (Sorsa et al., 2022). After periodontal/peri‐implant treatment, these levels decrease (Al‐Majid et al., 2018; Sorsa et al., 1988). A chairside point‐of‐care (PoC) test for the detection of aMMP‐8 in oral fluids has been developed for periodontitis and peri‐implantitis (PerioSafe® and ImplantSafe®, respectively). In our previous short report, we have shown statistically significant differences of aMMP‐8 levels between healthy, mucositis, and peri‐implantitis groups and a significant correlation of increasing probing depths of the sampled site and aMMP‐8 levels (Xanthopoulou et al., 2022).

Azurocidin, also called cationic antimicrobial protein 37 or human heparin‐binding protein, is a 29‐kDa glycoprotein derived from PMNs with antimicrobial activity, which binds to lipopolysaccharides due to its' cationic nature and disrupts the structure of the outer membrane of Gram‐negative bacteria (Almeida et al., 1996; Wilde et al., 1990). Azurocidin has been shown to be upregulated in periodontal disease (Guzman et al., 2018) and not to be present after periodontal treatment using high‐throughput proteomic analysis (Guzman et al., 2018). So, azurocidin can serve as a candidate biomarker for periodontal and peri‐implant diseases.

The aim of the study was to investigate and compare the potential of testing for aMMP‐8 by a quantitative PoC, chairside, lateral flow immunotest, and azurocidin in peri‐implant sulcular fluid (PISF) as biomarkers for the presence or absence of peri‐implant diseases in implant‐supported dental prostheses.

## 2 MATERIALS AND METHODS

### 2.1 Study design and inclusion/exclusion criteria

The study was designed as a cross‐sectional study. Eighty participants were recruited from the Department of Periodontology and Implant Biology and the Department of Prosthodontics of the Dental Faculty, School of Health Sciences, Aristotle University of Thessaloniki and constituted a convenience sample. Participants signed an informed consent form, and the study was approved by the Ethical Committee of the School of Dentistry, Aristotle University of Thessaloniki (#10/26.02.2020). Patients participating in this study should have at least one implant functionally loaded for at least 1 year.

Criteria for inclusion in the study were the absence of systemic immunomodulating diseases, infectious diseases (HIV/HBV/HCV infection), periodontal treatment or use of antibiotics for at least 6 months ago, and the presence of an implant with a functional load for at least 1 year.

### 2.2 PISF sampling and analysis

Before clinical examination, two samples were taken from PISF for analysis of aMMP‐8 and azurocidin levels from one implant in the oral cavity. If the patient had more than one dental implant, only one implant was randomly chosen, and two PISF samples were taken at the mesio‐buccal site with a time interval of 10 min.

In brief, the sampling sites were isolated, air dried, and isolated with cotton rolls, supra‐gingival biofilm was gently removed, and then paper strips (Periopaper®) were inserted into the peri‐implant sulcus/pocket until mild resistance felt and left in place for 30 s. Strips that were visually contaminated with blood or saliva were discarded (Nalmpantis et al., 2020).

The first sample was analyzed for aMMP‐8, using the ImplantSafe® diagnostic Kit (Dentognostics®, GmbH) according to the manufacturer's instructions (Golub et al., 2020; Lähteenmäki et al., 2020; Sorsa et al., 2020b). The test was used for both qualitative and quantitative analysis. The result was visible as blue lines on the dipstick. A single blue line indicated aMMP‐8 levels less than 20 ng/mL (negative) and two blue lines at levels higher than 20 ng/mL (positive) (Golub et al., 2020). Furthermore, the result on the dipstick was documented with a photograph. Levels of aMMP‐8 were assessed quantitatively in ng/mL by the digital reader Oralyzer® that accompanies ImplantSafe®.

The second PISF sample which was taken from the same site after 10 min was immediately frozen and stored at −80°C, and when all samples were collected, they were analyzed for azurocidin levels with enzyme‐linked immunoassay (Human AZU 1 ELISA Kit, Elabscience www.elabscience.com) according to the manufacturer's instructions. ELISA values were transformed and expressed for comparisons in pg/30 s, sample.

### 2.3 Clinical assessments

The appropriate clinical examination was performed, which included the following measurements: full‐mouth plaque score (FMPS), percentage of sites positive on BoP, PPD, and PAL.

- BoP: presence (+) or absence (−) of bleeding in percentage (%) 30 s after probe insertion in the peri‐implant pocket.
- FMPS: full‐mouth plaque score.
- PPD: the distance from the mucosa margin to the bottom of the peri‐implant pocket.
- PAL: the distance from the prosthetic crown shoulder to the bottom of the sulcus or the peri‐implant pocket.

All measurements were recorded at six sites per implant with a 15‐mm scale periodontal probe and graded per 1 mm (Hu‐friedy® CP‐12, #30).

All the examinations were performed by the same calibrated examiner (V.X) (Guzman et al., 2018).

X‐ray imaging of the implants followed the clinical examination. The x‐ray was taken using a digital x‐ray imaging system with phosphor plates (SCANORA 37, Software SOREDEX). For each case, all x‐rays were taken using the same irradiation time which corresponds to the time indicated on the x‐ray machine depending on the dental group to which the implant belongs. The type of implants and their prosthetic restoration were also evaluated.

### 2.4 Statistical analysis

Comparisons of azurocidin levels and clinical parameters were analyzed by the Kruskal–Wallis test and pairwise post hoc Dunn‐Bonferroni test. The diagnostic ability of aMMP‐8 (ng/mL) and azurocidin (pg/30 s sample) was investigated by the receiver operator curve (ROC) analysis and the calculated area under the curve (AUC).

The optimal cutoffs for each biomarker were calculated using the ROC curves based on the Youden index. The quality of classification of each biomarker based on the optimal cutoff was evaluated by diagnostic sensitivity (Se), specificity (Sp), the percentage of false negatives (FNs) and false positives (FPs), and test accuracy (Acc).

## 3 RESULTS

No differences were observed regarding age or sex distribution between patients with healthy implants, peri‐implant mucositis, or peri‐implantitis (age range: 52.5 ± 12.9, 57.6 ± 10, and 62 ± 6.7 years, respectively, *z*‐test for column proportion with Bonferroni correction, *p* > .05). Clinical data from the investigated implants and their comparisons are displayed in Table 1.

**Table 1:** Clinical parameters of the implant included in the study.

Implant	PL (%) M ± *SD*	PPD (mm) M ± *SD*	PAL (mm) M ± *SD*	BoP (%) M ± *SD*
Healthy implant (*n* = 27)	0.10 ± 0.23 (a)	3.11 ± 0.43 (a)	3.29 ± 0.60 (a)	0.00 ± 0.00 (a, b)
Peri‐imlant mucositis (*n* = 41)	0.22 ± 0.36 (b)	3.21 ± 0.31 (b)	3.28 ± 0.32 (b)	0.65 ± 0.20 (a)
Per‐implantitis (*n* = 12)	0.48 ± 0.42 (a, b)	5.87 ± 1.90 (a, b)	6.19 ± 1.98 (a, b)	0.88 ± 1.64 (b)

*Note*: Statistically significant differences in clinical parameters between groups are shown with the same letter (Dunn‐Bonferoni test after a significant Kruskal–Wallis test, *p* < .05).

Abbreviations: BoP, bleeding on probing; Μ, mean; n, number; PAL, probing attachment level; PL, plaque; PPD, probing pocket depth; *SD*, standard deviation.

Clinical parameters of periodontal characteristics of the implant included in the study displayed statistically significant differences for PL, PPD, and PAL between patients with healthy implant and with peri‐implantitis and between patients with peri‐implant mucositis and peri‐implantitis. For BoP, statistically significant differences were found between patients with healthy implant and with peri‐implant mucositis and healthy implant and peri‐implantitis (Dunn‐Bonferoni test after a significant Kruskal–Wallis test, *p* < .05) but not between the mucositis and peri‐implantitis groups.

Data‐concerning levels of aMMP‐8 and azurocidin in PISF are presented in Table 2. Statistically significant differences between patients with healthy implant and peri‐implant mucositis, healthy implant and peri‐implantitis, and peri‐implant mucositis and peri‐implantitis are depicted (Dunn‐Bonferoni test after a significant Kruskal–Wallis test, *p* < .05), but no differences were observed at any comparison for azurocidin levels (Kruskal–Wallis test, *p* > .05).

**Table 2:** Levels of aMMP‐8 and azurocidin of the investigated implants.

Implants	aMMP‐8 (ng/mL) mean ± *SD*	Azurocidin pg/30 s sample mean ± *SD*
Healthy implants (*n* = 27)	13.65 ± 7.18 (a, b)	33.16 ± 52.88
Peri‐implant mucositis (*n* = 41)	32.22 ± 21.20 (a, c)	52.86 ± 61.00
Peri‐implantitis (*n* = 12)	73.07 ± 43.93 (b, c)	35.53 ± 58.86

*Note*: Statistically significant differences in levels of aMMP‐8 between groups are shown with the same letter (Dunn‐Bonferoni test after a significant Kruskal–Wallis test *p* < .05). No differences were observed for azurocidin levels between groups (Dunn‐Bonferoni test after a significant Kruskal–Wallis test *p* > .05).

Abbreviations: aMMP‐8, active matrix metalloproteinase‐8; n, number; *SD*, standard deviation.

**Figure 1:**
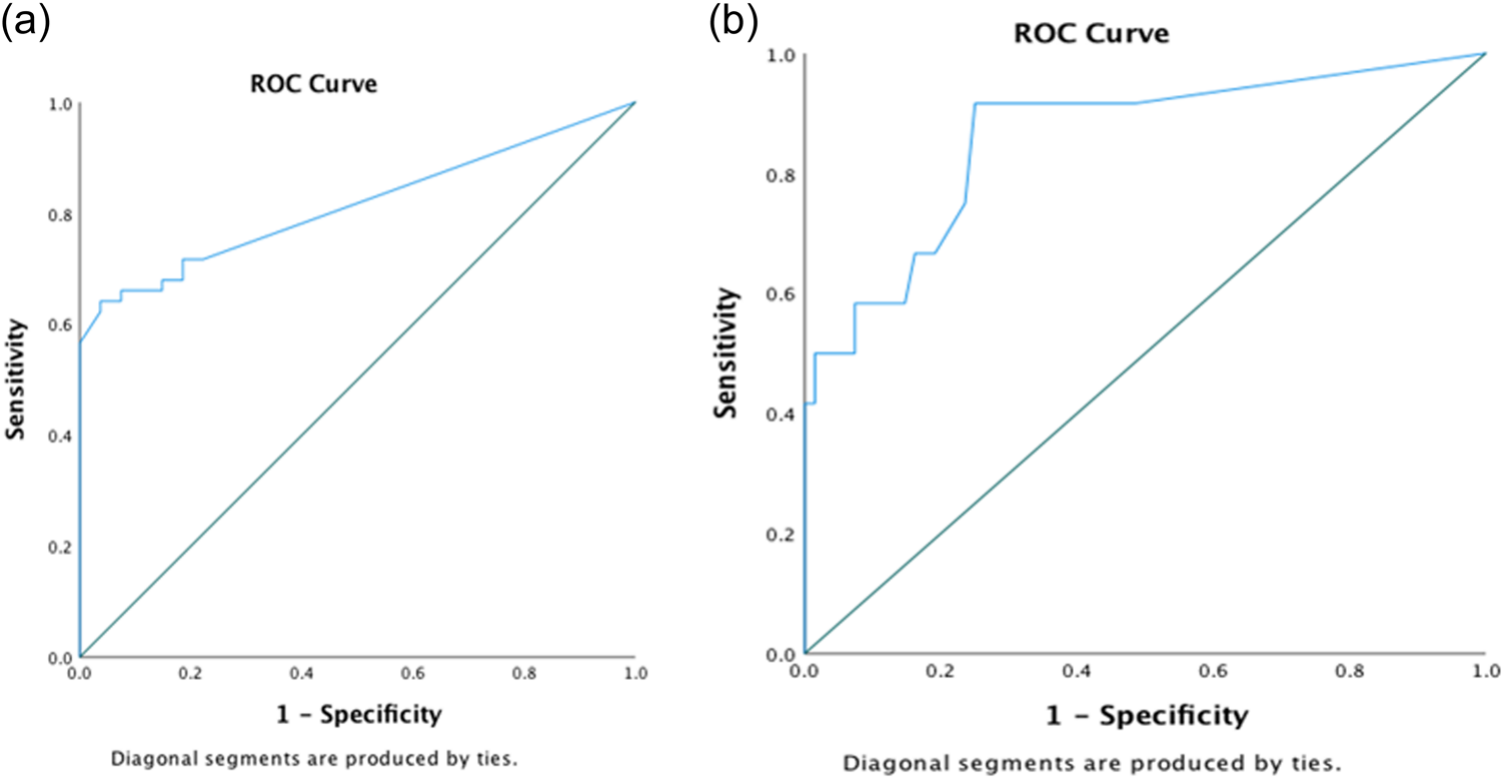
ROC analysis of the diagnostic ability of ImplantSafe®/ORALyzer® combination to discriminate (a) peri‐implantitis cases from healthy and mucositis and (b) healthy from mucositis and peri‐implantitis cases. ROC, receiver operator curve.

ROC was calculated to analyze the diagnostic ability of ImplantSafe®/ORALyzer® combination to discriminate healthy from mucositis and peri‐implantitis, with a cutoff value 20 ng/mL. The aMMP‐8 test with a threshold of 20 ng/mL has a sensitivity of 71.7% and a specificity of 77.8% to identify healthy implants. AUC was calculated and found to be 0.814, and the accuracy of the method reaches 73.8% (Figure 1b).

ROC was also calculated to analyze the diagnostic ability of ImplantSafe®/ORALyzer® combination to discriminate peri‐implantitis cases from healthy and implants with mucositis, again with a cutoff value 20 ng/mL. The aMMP‐8 test at 20 ng/mL has a high sensitivity for peri‐implantitis (91.7%) but a low specificity (51.5%). AUC was calculated and found to be 0.860 (Figure 1a). It is reminded that levels of aMMP‐8 above the cutoff value of 20 ng/mL can also be detected qualitatively and visually by two blue lines on the dipstick.

**Figure 2:**
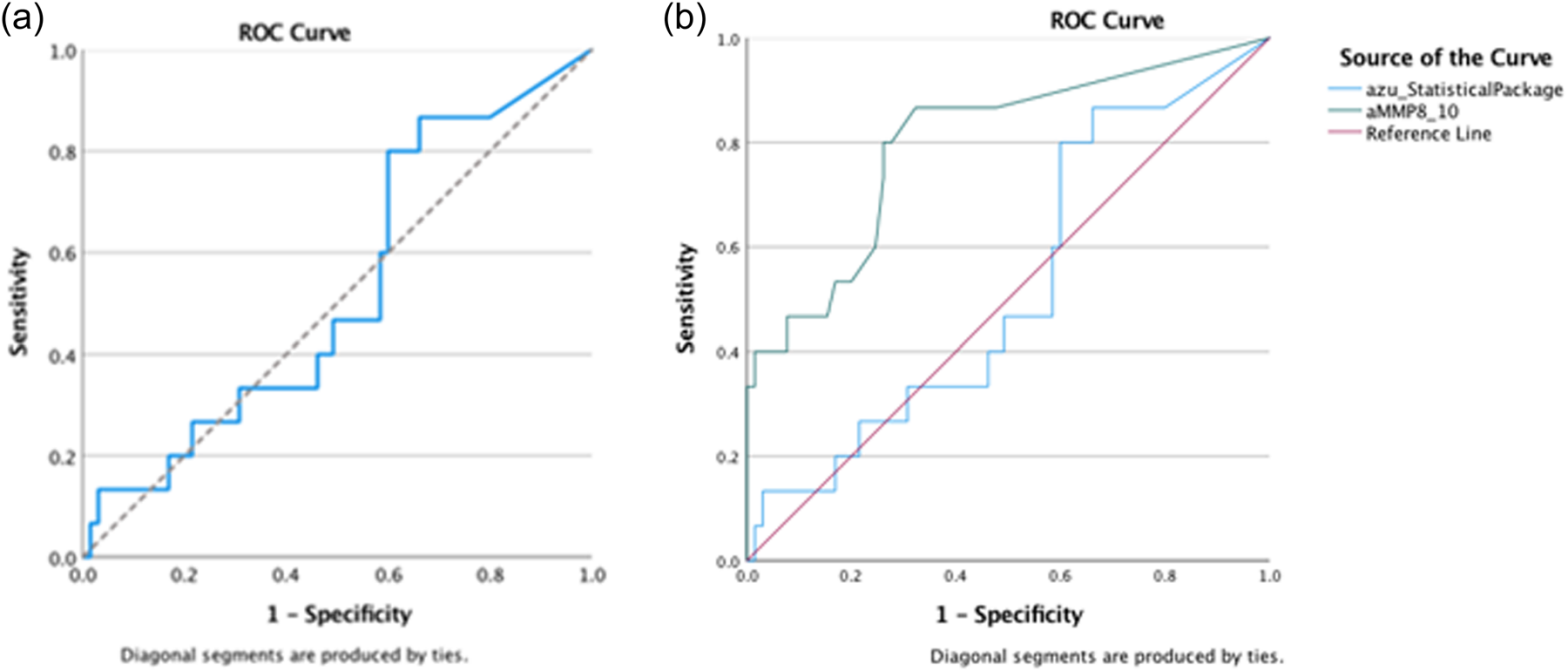
(a) ROC analysis regarding sensitivity and specificity of azurocidin. (b) Comparison of diagnostic performance of aMMP‐8 and azurocidin. aMMP‐8, active matrix metalloproteinase‐8; ROC, receiver operator curve.

The sensitivity of the assay (detection in samples with PD ≥ 5 mm and at least one site with BoP) for presence of azurocidin in PISF was 80%, but the specificity was 40%. The diagnostic accuracy of the method was estimated to reach 47.5%. Cutoff Youden's index for azurocidin was 11.7781 ng/mL (Figure 2a). When comparing azurocidin performance with aMMP‐8 for the same parameters, thus the ability to detect implants with PPD ≥ 5 mm and at least one site with BoP, ROC curves which integrated qualitative data from PISF samples provided more precise data regarding their diagnostic performance. It was displayed that aMMP‐8 for a Youden's index cutoff value of 32.15 ng/mL has greater sensitivity to identify peri‐implantitis patients than azurocidin. AUC for aMMP‐8 was calculated to reach the level of 0.798 and for azurocidin the level of 0.525 (Figure 2b).

When seeking for statistical correlations between clinical parameters and aMMP‐8 levels, correlation at the 0.01 level between aMMP‐8 and PPD, PAL, and BoP and correlation at the 0.05 level between aMMP‐8 and plaque were observed by applying the Spearman's rho test. No such correlations between azurocidin and clinical parameters of the investigated implant at the 0.05 level were observed by applying the Spearman's rho test.

**Figure 3:**
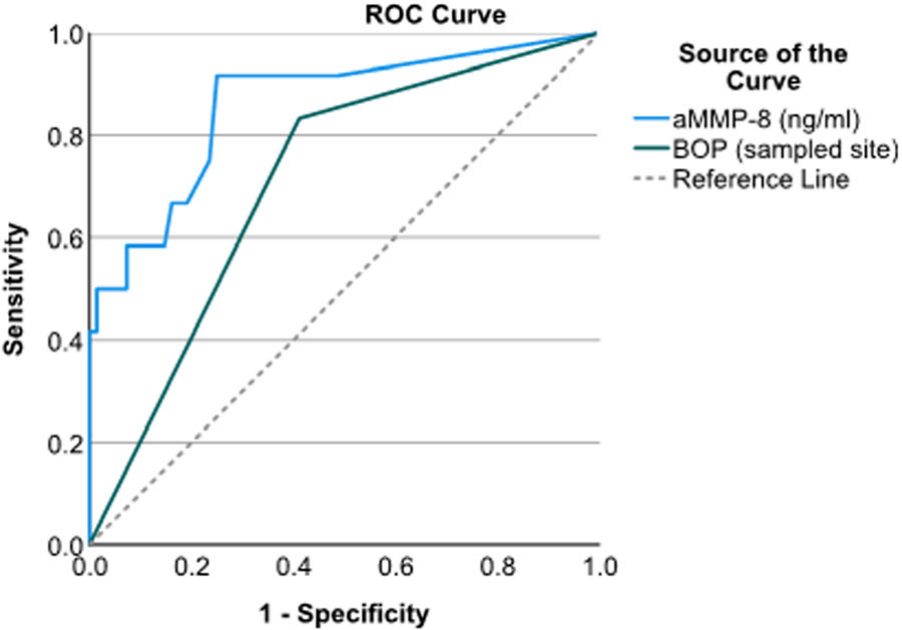
ROC analysis of aMMP‐8 (ng/mL) and bleeding on probing (BoP). aMMP‐8 has great sensitivity to identify peri‐implantitis patients from healthy and those with peri‐implant mucositis compared to the presence of BoP. aMMP‐8, active matrix metalloproteinase‐8; ROC, receiver operator curve.

Furthermore, the diagnostic potential of aMMP‐8 (ng/mL) and BoP were measured from the same site to identify peri‐implantitis (*n* = 80). Optimal cutoffs were defined by the Youden's index from the ROC curves. In specific, data presented include odds ratio, sensitivity, specificity, false negative, false positive, accuracy, and Matthew's correlation coefficient (Table 3 and Figure 3). It is shown that for above a cutoff value of 33.7 ng/mL of aMMP‐8 the accuracy of the test reaches 77.5% compared to 62.5% of BoP from the same site.

**Table 3:** Diagnostic potential of aMMP‐8 (ng/mL) and bleeding on probing (BoP) measured from the same site to identify peri‐implantitis.

Test	Peri‐implantitis (*N* = 12)	Healthy + mucositis (*N* = 68)	AUC (95%)	OR	Se	Sp	FN	FP	Acc	MCC
aMMP8 > 33.7 ng/mL	11	17	0.860 (0.734–0.985)	22.56	91.7%	75.0%	1.9%	60.7%	77.5%	0.50
aMMP8 < 33.7 ng/mL	1	51								
BOP positive (sampled site)	10	28	0.711 (0.564–0.858)	5.97	83.3%	58.8%	4.8%	73.3%	62.5%	0.30
BOP negative (sampled site)	2	40								

Abbreviations: Acc, accuracy; aMMP‐8, active matrix metalloproteinase‐8; AUC, area under the curve; FN, false negative; FP, false positive; MCC, Matthew's correlation coefficient; OR, odds ratio; Se, sensitivity; Sp, specificity.

ROC analysis of aMMP‐8 (ng/mL) and BoP (yes/no) measured from the same site also displayed that aMMP‐8 levels have great sensitivity to identify peri‐implantitis patients from healthy and those with peri‐implant mucositis compared to BoP for concentrations of aΜΜP‐8 ≥33.7 ng/mL (Figure 3).

## 4 DISCUSSION

In the present study, the diagnostic potential of an aMMP‐8 PoC test and azurocidin to discriminate peri‐implant conditions before clinical and radiographic assesments was investigated. Two PISF samples were collected, from one implant site of each participant. The first sample was quantitatively analyzed, chairside, by the ImplantSafe/OraLyzer® system for assessment of aMMP‐8 levels in ng/mL, chairside, and azurocidin levels were quantified using the ELISA technique from the second sample, when all samples were collected.

According to depicted data, the groups were comparable in terms of age and gender. It was observed that levels of aMMP‐8 in PISF were significantly higher in implants with peri‐implantitis when compared to those in healthy implants or implants with mucositis, suggesting destruction of peri‐implant tissues by collagenolysis. Low aMMP‐8 levels (20 ng/mL) in PISF were clearly linked to healthy implants.

Moreover, statistical analysis displayed a statistically significant correlation between increasing levels of aMMP‐8 in PISF and increasing probing depth, PAL, BoP, and plaque scores in the sampled site. These findings are in agreement with previous studies that have shown direct correlation between aMMP‐8 levels and periodontal/peri‐implant clinical parameters, indicating that this biomarker may potentially serve to diagnose and monitor the course of the disease (Alassiri et al., 2018; Kivelä‐Rajamäki et al., 2003a; Lähteenmäki et al., 2020; Leppilahti et al., 2014; Räisänen et al., 2019; Sorsa et al., 2020b) and identify sublinical inflammation, while this strong correlation was not observed between the levels of the total enzyme (both active and latent forms) of MMP‐8 (Lähteenmäki et al., 2022).

A main difference between the present and previous studies is the fact that clinical diagnosis in 80 consequent patients which fulfilled the inclusion criteria was set after the collection of PISF, while in other studies, addressing the diagnostic potential of aMMP‐8 the patients was already stratified according to their peri‐implant condition (healthy, mucositis, peri‐implantitis). Therefore, due to the design of the study, only 12 peri‐implantitis cases were included. Despite this caveat, our findings are in strong agreement with previous studies, which have also unanimously shown higher levels of aMMP‐8 in PISF from peri‐implantitis cases, compared to healthy ones (Alassiri et al., 2018; Kivelä‐Rajamäki et al., 2003b; Lähteenmäki et al., 2020; Sorsa et al., 2020b; Thierbach et al., 2016).

Previous studies have used various definitions of peri‐implant disease, and therefore, results are not easy to compare. The main criteria used in studies for peri‐implantitis have been based on BoP, PD ≥ 5 mm, and crestal bone loss of ≥2 mm, and for peri‐implant health, these have been based on PPD ≤ 5 mm and absence of BoP (Renvert et al., 2018). In the present investigation, the criteria and definitions of the 2018 classification of peri‐implant conditions have been applied, but despite the promising findings, the confined number of participants can be considered a limitation.

In the 2018 classification of periodontal diseases, the perspective of applying biomarkers is stated (Tonetti et al., 2018). Alike, biomarkers can be potentially integrated for peri‐implant disease assessment in the corresponding classification system (Berglundh et al., 2018). A number of studies concerning periodontal diseases have provided promising results of aMMP‐8 as an accessory tool in diagnosing periodontitis together with the common clinical methods (Alassiri et al., 2018; Chaparro et al., 2021; Sorsa et al., 2020b). Likewise, previous studies and the present study demonstrate and further extend that aMMP‐8 similarly can serve as a biomarker in the new classification system of peri‐implant diseases (Alassiri et al., 2018; Golub et al., 2020; Lähteenmäki et al., 2020; Sorsa et al., 2020b; Thierbach et al., 2016).

Certain of the above‐mentioned studies also depicted superiority of the aMMP‐8 chairside test compared to other technologies such as IFMA (Alassiri et al., 2018; Lähteenmäki et al., 2020), BoP (Sorsa et al., 2020b), total MMP‐8, calprotectin/interleukin (IL‐6) analyzed by ELISA, aMMP‐8 analyzed by western immunoblot, and MMP‐2/MMP‐9 analyzed by gelatin zymography (Lähteenmäki et al., 2020). When the threshold is set at the level of 20 ng/mL, as shown to be effective to discriminate periodontal breakdown, the sensitivity of the assay reaches 91.7% with a specificity 51.5% to identify healthy cases. Thus, levels <20 ng/mL, i.e., visually negative ones, can well be regarded as the biomarker at peri‐implant health as previously recorded for periodontally healthy sites (Öztürk et al., 2021; Sorsa et al., 2022).

In our previous study, it was shown that when setting a threshold of 32.15 ng/mL, this POC test can detect implants with at least one site with PD ≥ 5 mm together with at least one site with BoP with sensitivity and specificity 86.7% and 67.7%, respectively (Xanthopoulou et al., 2022). When setting the threshold level at 33.7 ng/mL, the sensitivity of the assay reaches 91.7% and the specificity 75% to identify peri‐implantitis cases. Both sensitivity and specificity of the assay were higher than the ones of BoP for the same cases (sensitivity: 83.3%, specificity: 58%).

Therefore, when the threshold of aMMP‐8 is at 20 ng/mL, the test is able to discriminate healthy cases with 71.7% sensitivity and 77.8% specificity, and the clinician is aware that no subclinical inflammation is present with an accuracy of 73.8%. Values above this threshold could indicate that, albeit clinical criteria of health are met, the clinician and the patient should adopt a protocol of frequent follow‐ups to ensure peri‐implant healthy conditions. On the other hand, when aMMP‐8 levels are above 32.15 ng/mL, there is a strong possibility of identifying peri‐implantitis cases.

Concerning azurocidin levels in PISF, the present study did not show any statistically significant differences between the peri‐implant healthy, peri‐implant mucositis, and peri‐implantitis groups. Azurocidin as a biomarker in PISF showed sensitivity at a rate 80%, while it presented specificity at a rate 40%, percentage that increases the probability of error in the use of azurocidin as a biomarker for peri‐implant disease. Finally, the diagnostic accuracy of azurocidin, that is, its ability in differential diagnosis of peri‐implant disease (at least one 5 mm PPD together with at least one site with BoP on the implant) reached the percentage of 47.5% and was inferior compared to aMMP‐8 in discriminating peri‐implant conditions (AUC: 0.860/0.463, respectively). The above data, although derived from a limited number of cases, do not support the view that the presence or absence of azurocidin can be used as an accurate diagnostic tool for the presence or absence of peri‐implant disease.

To our knowledge, this is the first report in the literature investigating azurocidin as a biomarker in peri‐implantitis. Previous reports in the literature for azurocidin refer to periodontitis cases, and there is considerable variation in the methods used for collection, processing, and analysis of GCF or PISF samples among different research groups. In the present study, the standard protocol for sampling was followed, as it is reported in the review of Wassall and Preshaw, (2016). The PISF samples were collected before probing depth recording, stored dry with no buffer in the microtubes at −80°C for a maximum period of 6 months, and analyzed individually.

A main point that might account for this difference is the fact that in the studies of Choi et al. (2011), Guzman et al. (2018), and Nalmpantis et al. (2020), pooled GCF samples were collected. This method has the advantage of collecting a larger amount of liquid in units and at the same time absorbing more of its proteins and other components, in comparison to our study, in which the sample was taken from one site only. In addition, an important differentiation of the present study is the fact that the sample analyzed for azurocidin was taken from the same site 10 min after the aMMP‐8 sample. In the above mentioned studies, only one sample was collected and analyzed. It is known that there is continuous GCF flow, usually only a few microliters per hour, and it is accepted that intervals of 10 min or more should be sufficient for re‐establishing an equilibrium of GCF between two samples (Goodson, 2003). Therefore, for the purposes of the present study, it was decided that that the second sample will be collected 10 min after the first one. One might speculate that albeit data in the literature, there was a difference regarding the amount of GCF compared to the first sample. This fact might explain, at least in part, for the levels of azurocidin as determined in the present study.

Another noteworthy point is the storage conditions of the samples. In the systematic review of Barros et al. (2016), the advantage of quick freezing the samples in liquid nitrogen before storing are reported. Liquid nitrogen displaces the dissolved oxygen and protects it from reacting with molecular oxygen and oxidation. As a result, the components can be stored for a long time and remain stable without degradation by enzymes and without oxidation (Barros et al., 2016). In the study of Guzman et al. (2018) and Nalmpantis et al. (2020), the samples were quickly frozen in liquid nitrogen and then stored at constant temperature conditions −80°C for a maximum period of 6 months. In the present study, the samples were only immediately frozen and stored at −80°C for a maximum period of 6 months, and this may be another reason that might have partially affected levels of azurocidin in the samples.

Taken collectively, data from the present study provide encouraging evidence to establish and further extend the value of the chairside, PoC aMMP‐8 test, for identification of peri‐implant tissue health or inflammation. In addition, the ability of the test to predict future peri‐implant tissue breakdown, in longitudinal studies, should be investigated. Large‐scale studies which can also investigate the threshold of aMMP‐8 levels suitable for discriminating peri‐implant health and disease with high sensitivity and specificity are suggested.

## AUTHOR CONTRIBUTIONS

*Conceptualization*: Dimitra Sakellari and Timo Sorsa. *Methodology*: Dimitra Sakellari, Timo Sorsa, Ismo T. Räisänen, Vithleem Xanthopoulou. *Software*: Ismo T. Räisänen. *Validation*: Dimitra Sakellari, Timo Sorsa and Ismo T. Räisänen; Formal analysis, Ismo T. Räisänen. *Investigation*: Dimitra Sakellari, and Vithleem Xanthopoulou. Resources, Dimitra Sakellari, Timo Sorsa; Data Curation, Ismo T. Räisänen. *Writing—Original Draft Preparation*: Ismo T. Räisänen, Vithleem Xanthopoulou. *Writing—Review and Editing*: Dimitra Sakellari, Timo Sorsa. *Visualization*: Dimitra Sakellari, Timo Sorsa, Ismo T. Räisänen, Vithleem Xanthopoulou. *Supervision*: Dimitra Sakellari, Timo Sorsa. *Project Administration*: Dimitra Sakellari, Timo Sorsa. *Funding Acquisition*: Timo Sorsa, Ismo T. Räisänen.

## CONFLICT OF INTEREST STATEMENT

TS is the inventor of U.S. patents 1,274,416, 5,652,223, 5,736,341, 5,864,632, 6,143,476, and US 2017/0023571A1 (issued June 6, 2019), WO 2018/060553 A1 (issued May 31, 2018), 10,488,415 B2, and US 2017/0023671A1, Japanese Patent 2016‐554676 and South Korean Patent No. 10‐2016‐7025378. The remaining authors declare that the research was conducted in the absence of any commercial or financial relationships that could be construed as a potential conflict of interest.

## ETHICS STATEMENT

Participants signed an informed consent form, and the study was approved by the Ethical Committee of the School of Dentistry, Aristotle University of Thessaloniki (#10/26.02.2020).

## Data Availability

The data used to support the findings of this study are available from the corresponding author upon request.
